# Effect of Dietary Sugarcane Bagasse Supplementation on Growth Performance, Immune Response, and Immune and Antioxidant-Related Gene Expressions of Nile Tilapia (*Oreochromis niloticus*) Cultured under Biofloc System

**DOI:** 10.3390/ani11072035

**Published:** 2021-07-08

**Authors:** Chompunut Lumsangkul, Wanaporn Tapingkae, Korawan Sringarm, Sanchai Jaturasitha, Chinh Le Xuan, Supreya Wannavijit, Piyatida Outama, Hien Van Doan

**Affiliations:** 1 Department of Animal and Aquatic Sciences, Faculty of Agriculture, Chiang Mai University, Chiang Mai 50200, Thailand; chompunut.lum@cmu.ac.th (C.L.); wanaporn.t@cmu.ac.th (W.T.); korawan.s@cmu.ac.th (K.S.); lechinh864@gmail.com (C.L.X.); phoooooooooo10@gmail.com (S.W.); outamapiyatida@gmail.com (P.O.); 2 Science and Technology Research Institute, Chiang Mai University, 239 Huay Keaw Rd., Suthep, Muang, Chiang Mai 50200, Thailand; ja.sanchai@gmail.com

**Keywords:** sugarcane bagasse, Nile tilapia, growth performance, immune response, gene expressions

## Abstract

**Simple Summary:**

Supplementation of agriculture by-product as functional feed additives in combination with biofloc technology (a sustainable and environmentally friendly technology) has recently gained much attention in aquaculture. In the present study, sugarcane bagasse powder can possibly be applied as a feed additive to improve growth performance, immune response, and immune and antioxidant-related gene expression.

**Abstract:**

We investigated, herein, the effects of dietary inclusion of sugarcane bagasse powder (SB) on Nile tilapia development, mucosal and serum immunities, and relative immune and antioxidant genes. Fish (15.12 ± 0.04 g) were provided a basal diet (SB0) or basal diet incorporated with SB at 10 (SB10), 20 (SB20), 40 (SB40), or 80 (SB80) g kg^−1^ for 8 weeks. Our results demonstrated that the dietary incorporation of sugarcane bagasse powder (SB) at 20 and 40 g kg^−1^ significantly ameliorated FW, WG, and SGR as opposed to fish fed basal, SB10, and SB80 diets. However, no significant changes in FCR and survivability were observed between the SB supplemented diets and the control (basal diet). The mucosal immunity exhibited significantly higher SMLA and SMPA activities (*p* < 0.005) in fish treated with SB diets after eight weeks. The highest SMLA and SMPA levels were recorded in fish fed SB80 followed by SB20, SB40, and SB10, respectively. For serum immunity, fish fed SB incorporated diets significantly ameliorated SL and RB levels (*p* < 0.05) compared with the control. However, SP was not affected by the inclusion of SB in any diet throughout the experiment. The expression of IL1, IL8, LBP, GSTa, GPX, and GSR genes in the fish liver was significantly increased in fish fed the SB20 and SB10 diets relative to the basal diet fed fish (*p* < 0.05); whereas only the IL8, LBP, and GPX genes in the intestines were substantially augmented via the SB20 and SB80 diets (*p* < 0.05). IL1 and GSR were not influenced by the SB incorporated diets (*p* > 0.05). In summary, sugarcane bagasse powder (SB) may be applied as a feed additive to improve growth performance, immune response, and immune and antioxidant-related gene expression in Nile tilapia.

## 1. Introduction

The aquaculture industry produces upwards of half of the globe’s seafood and is responsible for a dramatic expansion of human food production [1,2]. Nile tilapia is one of the most widely cultivated fish worldwide, due to its flexibility and high economic value [3,4]. Nevertheless, the super-intensification of tilapia farming has imposed serious strains on several cultured ecosystems and has increased susceptibility to diseases, especially bacterial infections [5,6], giving rise to sizable death rates and significant property damage [7]. Antibiotics have been commonly used in the past century to prevent and treat bacterial infections all over the world [8]. Antibiotic therapies, on the other hand, have promoted the development of antimicrobial bacteria and the deterioration of cultivated ecosystems [9]. Contrastingly, feed cost in super-intensive fish farming can account for up to 70% of overall operational costs [10,11] and is often performed solely with rations [12]. As protein-based feedstuff in aquafeed adds significantly to the cost, the application of non-protein feedstuff, including lipids and carbohydrates, can diminish the use of protein as an energy source [13,14]. In this regard, to keep pace with considerable developments taking place in the aquaculture industry, advance manufactured technologies, especially concerning cost-effective feed and environmentally friendly cultured systems, are needed.

The application of functional supplements has grown in popularity in fish farming [15,16]. Agricultural co-products, in this sense, provide a potential source of dietary fibers, acting as prebiotics, that may be used as biomedical compounds to cure symptoms associated to intestinal alteration [17]. Sugarcane bagasse (SB) is one option for this commodity. SB is an abundantly produced by-product from the sugar-making process after the sugarcane juice has been extracted [18], and is estimated to account for about 78.04 thousand metric tons yearly [19]. Due to the deficiency of profitable treatments and recirculation manners, most SB is burned, discarded, or utilized as pulp [20,21,22]. As with many other agricultural by-products, SB is rich in polysaccharides, which include cellulose and hemicellulose [23,24,25,26]. SB hemicellulose is mostly made up of xylan, which is of special concern, given that xylooligosaccharides (XOS) represent a potential prebiotic compound [18,27,28,29]. Therefore, employing such a by-product would generate a value-added element to this manufacturing waste and offer a valuable and much-needed raw resource for pharmaceutical and aquacultural industries [30].

Fish, like many vertebrates, possess a complicated immune system, including innate and specific immune responses. The first layer of defense includes epidermal mucus that is varied biologically in its activated components, including lectins, lysozymes, antibacterial peptides, and immunoglobulins [31,32,33]. Furthermore, cytokines, as a part of cell-mediated immune response, are simple water-soluble polypeptides, which are secreted by several immune cells in response to antigens [34]. Lipopolysaccharide binding protein (LBP) gene is involved in the acute-phase immunologic response to bacterial infections in Nile tilapia against *Streptococcus agalactiae* and *Aeromonas hydrophila* [35]. On the other hand, the glutathione (GSH)-relative antioxidant system plays a key role in the intercellular defense mechanism counteracting oxidative stress, which is involved of GSH and its related enzymes, including glutathione peroxidase (GPx), glutathione reductase (GR), and glutathione S-transferase (GST) [36,37].

Biofloc technology (BFT) has become a profitable, ecologically responsible, and sustainable aquaculture system [38,39,40,41]. This technology is primarily founded on the principle of waste nutrient recycling, especially nitrogen, into microbial biomass that can be used in situ by the culture fish and shellfish or be harvested and processed into feed ingredients [42]. As demonstrated in previous research, biofloc technology has shown numerous positive effects on water quality, productivity, immune response, and disease prevention in aquaculture [38,39]. Prebiotics derived from agricultural by-products, on the other hand, play an equally essential role in aquaculture farming [43,44]. The addition of these products to the biofloc system is intended to enhance favorable microorganisms, not only in the cultured water but also in the host’s gut to combat the potentially harmful bacteria. Recent studies have been undertaken in accordance with this theory, in which biofloc water was found to significantly enhance water quality, the host’s performance, immune response, and disease resistance [45,46]. The effects of SB within the biofloc represents a novel and multidisciplinary strategy that has yet to be thoroughly explored through research. We speculated that the symbiotic relationship between SB powder and the biofloc system may strengthen Nile tilapia’s health and performance. The present study, thereby, investigated the impact of SB on performance, non-specific immune response, and relative immune and antioxidant gene expressions of Nile tilapia raised in the biofloc system.

## 2. Materials and Methods

### 2.1. Sugarcane Bagasse Powder Preparing

Sugarcane bagasse was collected from a local market, oven-dried for 48 h at 60 °C, pulverized, sieved through a 100-mesh screener, and then retained at 4 °C for further use.

### 2.2. Diets Description

Five trial diets were developed with the inclusion of SB at different rates: SB0, the control (0 g kg^−1^), SB10 (10 g kg^−1^), SB20 (20 g kg^−1^), SB40 (40 g kg^−1^), and SB80 (80 g kg^−1^) (Table 1). Feedstuffs were mixed and combined; then oil and distilled water were appended to make the dough. The product was then converted into pellets, then dehydrated at 50 °C to reach ~10% moisture, and preserved in bags at 4 °C.

### 2.3. Experimental Design

Nile tilapia were purchased from Chiang Mai Patana Farm and distributed in cages. In the adaptation period, fish were fed the control diet for two weeks. Their internal organs and gills were checked regularly by a light microscope to determine their health status. Thereafter, three hundred fish with an average weight of 15.12 ± 0.04 g were randomly dispersed into 15 tanks (150 L) and provided diets reiterated in triplicates. Twenty fish were stocked per tank, and the fish were fed to satiation twice daily, at 8:30 a.m. and 4:30 p.m., under a photoperiod of 12:12 h of darkness and light.

### 2.4. Biofloc Water Preparation

The tanks were prepared as the BF source of inoculants 3 weeks before the trial. To prepare the floc water, 2 g wheat flour, 400 g salt (400 g per tank), 5 g dolomite, and 5 g molasse were added to each tank. During the experimental period, the C:N ratio was maintained at 15:1 by adding molasses (40% C) as a carbon source, according to Avnimelech [47]. The C:N ratio was schematically computed based on the leftover nitrogen level in each tank, as well as the contribution of the diet [48]. Molasse was added daily, two hours post-feeding.

### 2.5. Samples Preparation

The mucus of the skin was collected, as described by Khodadadian Zou, Hoseinifar, Kolangi Miandare, and Hajimoradloo [49], after four and eight weeks of feeding. Briefly, fish were anesthetized with clove oil and smoothly massaged in a bag containing 50 mM NaCl. Subsequently, a sterile tube was used to centrifuge the solution at 1500 g at 4 °C for ten minutes. Afterward, supernatant (500 µL) was collected and kept in a freezer for further analysis.

The serum from blood samples was separated, as described in our previous studies [50,51] and preserved at −20 °C for further analyses. Briefly, blood (1 mL) was collected via the caudal vein of each fish using a 1mL syringe and immediately released into 1.5 mL Eppendorf tubes without anticoagulant. The blood samples were then led to clot at room temperature for one hour and stored in a refrigerator (4 °C) for four hours. After that, the samples were centrifuged at 1500× *g* for five minutes at 4 °C, and the anticipated serum was gathered using a micro-pipette and stored at −80 °C for further evaluation.

Leukocytes were prepared following the technique described in previous studies [50,51]. Briefly, one milliliter of blood was withdrawn from each fish at a rate of three fish per replication and then transferred into 15 mL tubes containing 2 mL of RPMI 1640 (Gibthai, Bangkok, Thailand). This mixture was then carefully inserted into 15mL tubes, containing 3 mL of Histopaque (Sigma, St. Louis, MO, USA). These tubes were then centrifuged at 400 g for 30 min at room temperature. Upon completion, a buffy coat of leucocyte cells that drifted to the top of the Histopaque was carefully collected using a Pasteur pipette, and released into sanitized 15 mL tubes, after which 6mL of phosphate buffer solution (PBS: Sigma-Aldrich, St. Louis, MO, USA) was added to each tube and gently aspirated. The cells in these tubes were washed twice by centrifugation at 250 g for ten minutes at room temperature to remove any residual Histopaque. The cells obtained were then re-suspended in the PBS and adjusted to the numbers of cells required to evaluate phagocytic and respiratory burst activities.

### 2.6. Immunological Parameters and Growth Performance

Lysozyme activity was detected according to Parry, Chandan, and Shahani [52] and presented as µg mL^−1^. Briefly, 25 µL of undiluted serum and 100 µL of skin mucus from each fish was loaded onto 96-well plates in triplication. *Micrococcus lysodeikticus* (175 µL, 0.3 mg mL^−1^ in 0.1 M citrate phosphate buffer, pH 5.8) was then added to each well. The contents were rapidly mixed, and any changes in turbidity were measured every 30 s for five minutes at 540 nm and 25 °C via a microplate reader. The sample’s equivalent unit of activity was determined and compared with the standard curve, which was generated from the reduction of OD value vs. the concentration of hen egg-white lysozyme ranging from 0–20 µL mL^−1^ (Sigma Aldrich, St. Louis, MO, USA), and expressed as µg mL^−1^ serum.

Peroxidase measurements were determined as stated by Van Doan, Hoseinifar, Dawood, Chitmanat, and Tayyamath [53]. Briefly, 5 µL of undiluted serum or skin mucus from each fish was placed on 96-flat-bottomed-well plates in triplicate. Then, 45 µL of Hank’s Balanced Salt Solution (without Ca^+2^ or Mg^+2^) was added to each well. Afterward, 100 µL of solution (40 mL of distilled water + 10 µL of H_2_O_2_, 30%; Sigma Aldrich + one pill of 3,3′,5,5′-tetramethylbenzidine, TMB; Sigma Aldrich) was then added to each well. When the reaction color turned blue, after 30 to 60 s, a 50 µL solution of 2M H_2_SO_4_ was immediately added to each well. The optical density was then read at 450nm via a microplate reader (Synergy H1, BioTek, Winooski, VT, USA). Samples not containing serum or skin mucus were considered to be blanks. A single unit was defined as the amount that produces an absorbance change, expressed as units (U) mL^−1^ of serum or mucus through the following equation: Peroxidase activity = [absorbance of the sample] − [absorbance of blank containing all solution without serum or mucus sample].

Respiratory burst activity was determined according to the protocol described by Secomebs [54], and growth parameters utilized the equations of Doan, Hoseinifar, Jaturasitha, Dawood, and Harikrishnan [55]. Briefly, 175 µL PBS cell suspension at a concentration of 6 × 10^6^ cells mL^−1^ was loaded into the 96 well plates in triplication. Then, 25 µL of nitro blue tetrazolium (NBT) at a concentration of 1mg mL^−1^ was added to each well and incubated for two hours at room temperature. Later, the supernatant was carefully discarded from each well, and 125 µL of 100% methanol was then added into each well for five minutes to fix the cells. After that, 125 µL of 70% methanol well^−1^ were added into each well, twice, for clean-up. The plates were then dried for thirty minutes at room temperature. Then, 125 µL of 2N KOH and 150 µL of DMSO were added to each well. Afterward, the plates were measured at 655 nm via microplate-reader (Synergy H1, BioTek, USA), according to the following: Spontaneous O_2_^-^ production = [absorbance NBT reduction of the sample] − [absorbance of blank containing 125 µL of 2N KOH and 150 µL with no leucocytes].

### 2.7. Immune and Antioxidant-Related Genes Expression in Liver and Intestine

#### 2.7.1. Tissue Sampling

At the end of the experiment, three fish from each treatment were randomly selected for liver and intestine collection. Fish were dissected and their liver and intestine tissues (25–50 mg) were removed and transferred to a 1.5 Eppendorf tube containing 500 μL of Trizol (Invitrogen #1IV11-15596-026), then frozen at −80 °C until RNA extraction.

#### 2.7.2. RNA Extraction and cDNA Synthesis

The liver and intestine tissues were homogenized using pellet pestles (Sigma-Aldrich). Afterward, the samples were incubated at room temperature for 5 min, and then 100 μL of chloroform was added to each tube, and again incubated at room temperature for 2 min. The tubes were then centrifuged for 15 min at 12,000× *g* at 4 °C. After centrifugation, the aqueous phase containing the RNA was transferred to a new tube then extracted using an RNA extraction kit (Invitrogen, PureLink^TM^ RNA Mini Kit, Fair Lawn, NJ, USA) according to the manufacturer’s instructions. The extracted RNA was quantified using a spectrophotometer (NanoDrop^TM^ 2000, Thermo Scientific, Wilmington, NC, USA) at an absorbance ratio of 260–280 nm. cDNA was synthesized using an iScript^TM^ cDNA Synthesis Kit (BIO-RAD, Hercules, CA, USA) according to the manufacturer’s instructions. The primer sequences of IL1, IL8, LBP, GSTa, GPX, and GSR genes, as well as the 18S rRNA as a housekeeping gene, are displayed in Table 2.

#### 2.7.3. Quantitative PCR

The qPCR reaction was carried out by CFX Connect^TM^ Real-Time PCR System (BIO-RAD, Hercules, CA, USA) using the iTaq Universal SYBR Green supermix 2X (BIO-RAD, USA) and specific primers for individual gene (Table 2). The qPCR was performed in triplicate using 100 ng of cDNA, 400 mM of primers. Thermal cycling conditions were 95 °C for 30 s (holding stage); 40 cycles of 95 °C for 15 s, and 60 °C for 30 s (cycling stage); followed by 95 °C for 15 s; 60 °C for 60 s; and 95 °C for 15 s (melt curve stage). Changes in the expression levels of the above genes were measured using the 2^−ΔΔCt^ method and a standard curve [56].

### 2.8. Statistical Analysis

The differences in studied parameters of immune response, gene expression, and growth performance among diets were determined using one-way analysis of variance (ANOVA) and Duncan’s multiple range test via SAS software [57]. Significantly different mean values (*p* < 0.05) and other data are displayed as means ± SE.

## 3. Results

### 3.1. Growth Performance

As summarized in Table 3, the dietary incorporation of sugarcane bagasse powder (SB) at 20 and 40 g kg^−1^ significantly increased final weight (FW), weight gain (WG), and specific growth rate (SGR) in contrast to fish fed basal, SB10, and SB80 diets. However, no noticeable change in feed conversion ratio (FCR) between the SB treated and non-treated diets, except for the fish fed diet SB80, which produced a higher FCR level than the control (Table 3). Survival rates of Nile tilapia were not influenced by the SB-treated diets (*p* > 0.05).

### 3.2. Skin Mucus Immunity

Table 4 illustrates the effects of SB on skin mucosal immunity of Nile tilapia. Based on the results, skin mucus lysozyme (SMLA) and skin mucus peroxidase (SMPA) activities were significantly higher (*p* < 0.005) in fish treated with the SB diets after eight weeks. The highest SMLA and SMPA levels were recorded in fish fed SB80; followed by the SB20, SB40, and SB10 diets, respectively (Table 4).

### 3.3. Serum Immunity

The amount of lysozyme (SL) in the serum differed greatly between groups (Table 5). Fish fed an SB supplemented diet produced a better SL level (*p* < 0.05) in contrast to non-treated groups. The best results were observed in the SB80 diet at four weeks and in the SB40 diet at eight weeks. Similarly, the respiratory burst activity (RB) level significantly improved in fish fed the SB10 diet versus the control and other treated groups at 4 weeks post-feeding. No meaningful change in RB was observed in any group at either four- or eight-weeks post-feeding. Additionally, SP was not influenced by the incorporation of SB throughout the experiment.

### 3.4. Expression of Immune-Related and Antioxidant Genes

**Figure 1:**
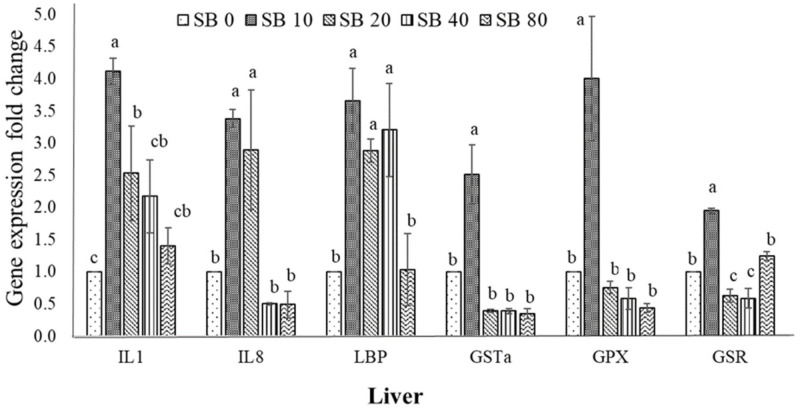
Expressions of immune (interleukin 1, IL1; interleukin 8, IL8; lipopolysaccharide binding protein, LBP) and antioxidant-related genes (glutathione S-transferase, GSTa; glutathione-disulfide reductase, GSR; glutathione peroxidase, GPX) in liver of Nile tilapia after treated with phenol rich. Three replicates. ^a,b,c^ Columns without the same superscripts differ (*p* < 0.05).

The effects of SB on the transcription levels of IL1, IL8, LBP, GSTa, GPX, and GSR in the livers of Nile tilapia are presented in Figure 1. The expression of IL1, IL8, and LBP significantly increased in the SB10 and SB20 diets relative to the basal diet-fed fish (*p* < 0.05). The highest upregulation of IL1 and IL8 was noticed in fish fed the SB10 supplemented diet. Similarly, significantly higher expression levels of GSTa, GPX, and GSR genes were found in fish fed the SB10 diet, as opposed to the other treated fish and un-treated fish (*p* < 0.05). No meaningful variations in IL1, IL8, LBP, GSTa, GPX, and GSR were found in fish fed the SB80 or basal diet (*p* > 0.05).

**Figure 2:**
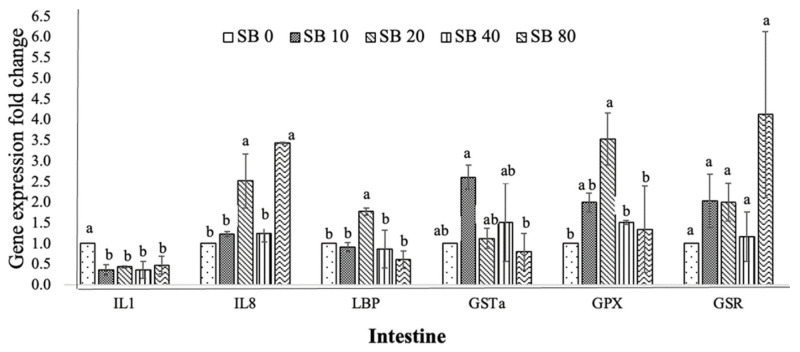
Expressions of immune (interleukin 1, IL1; interleukin 8, IL8; lipopolysaccharide binding protein, LBP) and antioxidant-related genes (glutathione S-transferase, GSTa; glutathione-disulfide reductase, GSR; glutathione peroxidase, GPX) in intestine of Nile tilapia after treated with phenol rich. Three replicates. ^a,b^ Columns without the same superscripts differ (*p* < 0.05).

Figure 2 illustrates the consequences of dietary SB on the transcription level of immune and antioxidant-related genes in the intestines of Nile tilapia. The expression levels of IL8, LBP, and GPX significantly increased in fish fed the SB20 and SB80 diets (*p* < 0.05). Nevertheless, no significant difference in IL8, LBP, and GPX expression levels was recorded in fish fed SB10, SB40, and SB80, respectively. IL1 and GSR were not influenced by the inclusion of SB supplements (*p* > 0.05).

## 4. Discussion

Fish skin mucus is the first layer of the innate immune system, which is released in cases of stress and outbreak [58,59,60]. The mucus consists of many biological molecules, such lysozyme, peroxidase, and bactericidal agents [61,62,63]. Our work indicated that fish fed SB diets had higher skin mucosal immunity than that of the control. Similar findings were reported in convict cichlid (*Amatitlania nigrofasciata*) [64]; gilthead seabream (*Sparus aurata*) [65]; hybrid tilapia (*Oreochromis niloticus* × *O. mossambicus*) [66]; common carp (*Cyprinus carpio*) [67]; Persian sturgeon (*Acipenser persicus*) [68]; Nile tilapia (*O. niloticus*) [69,70], and Siberian sturgeon (*Acipenser baerii*) [71]. Lysozyme is a proteolytic enzyme, which can kill bacteria by damaging their cell-wall and provoking other immune parameters, such as complement and phagocytosis activities [72]. On the other hand, respiratory burst, via motivation by foreign agents, is renowned for enhancing the oxidation levels in phagocytes, and is known to be an essential element in the fish defense mechanism [73,74]. Supplementation of SB in the present study increased lysozyme and respiratory burst activities. The findings were consistent with previous findings reported in gibel carp (*Carassius auratus gibelio*) [75]; hybrid grouper (*Epinephelus fuscoguttatus*♀ × *E. lanceolatus*♂) [76]; Nile tilapia (*O. niloticus*) [70]; and European seabass (*Dicentrarchus labrax*) [77]. The enhancements may be attributable to the flavonoids and phenolics in SB [78,79]. It is known that polyphenols can induce dendritic cells, have immunomodulatory effects on macrophages, and increase the proliferation of B and T cells [80].

Cytokines, which are primarily generated by white blood cells, play an essential part in modulating and linking non-specific and specific immune systems [81]. The present study indicated that IL-1 and IL-8 were significantly up-regulated in fish fed SB diets, particularly 10 g kg^−1^ SB. These are important cytokines of fish that aid in response to infected pathogens [82,83]. Our results were consistent with earlier studies in barramundi (*Lates calcarifer*) [84]; Nile tilapia (*O. niloticus*) [70]; Japanese flounder (*Paralichthys olivaceus*) [85]; rohu (*Labeo rohita*) [86], and European seabass (*Dicentrarchus labrax*) [77]. Lipopolysaccharide-binding protein (LBP) is a soluble acute-phase protein, which plays an essential role in the detection of bacterial elements that regulate cellular signals in phagocytic cells and is able to boost fish immune response [35,87,88]. Our findings are in line with studies reported in crucian carp (*Carassius carassius*) [89]; Atlantic salmon (*Salmo salar*) [90], and Nile tilapia (*O. niloticus*) [70]. The GPx and GSR enzymes work together in the glutathione protection mechanism to eliminate hydrogen peroxide (H_2_O_2_). GPx transforms H_2_O_2_ into water via oxidation of glutathione (GSH) to glutathione disulfide (GSSG). Once oxidized, GSH is revitalized by GSR via oxidizing reduction of NADPH [91]. Glutathione S-transferase (GST) is the phase II xenobiotic metabolic catalyst that utilizes phase I reactions to build bigger endogenic molecules, which are readily released through bile or kidney [92]. SB supplementation in the Nile tilapia diets substantially increased GST, GPX, and GSR transcription in fish livers, according to the present findings. The same conclusions were noted in Nile tilapia (*O. niloticus*) [93,94,95]; hybrid grouper (*Epinephelus lanceolatus* ♀ × *E. fuscoguttatus*♂) [96]; common carp (*Cyprinus carpio*) [97,98,99]; European seabass (*Dicentrarchus labrax*) [100], and rohu (*Labeo rohita*) [86]. The significantly enhanced immune response by Nile tilapia in the present study may be attributable to the bioactive compounds present in the SB, which contains a high amount of xylooligosaccharide, which is potentially prebiotic [18,101,102,103]. Xylooligosaccharide is known to enhance immune responses [104,105], and has been applied in aquafeed to stimulate fish immunity [106,107]. Moreover, the antioxidant properties have been accredited to the phenolic compounds content of SB, which scavenge oxidative activity [79,108,109,110]. Interestingly, IL-1, IL-8, LBP, GSTa, GPX, and GSR gene expressions in the liver were down-regulated in fish fed SB80 compared to SB10. This may be attributable to an overdose of immunostimulant administration, which generally resulted in immunosuppression [111]. Moreover, significantly up-regulated relative immune and antioxidant gene expressions were observed in fish liver, whereas no significant differences were determined in fish intestine. The difference in relative immune gene expression may be due to the difference in immune cell presence in each tissue. Fish intestine is immunologically active and armored with B cells, macrophages, granulocytes, and T cells, while in the liver, along with immunomodulatory and immune suppression genes, non-specific molecules, such as acute phase protein, complement components, and anti-microbial peptides, which could release from bile to intestinal mucus, were found to be of great importance for basic function [112]. In terms of antioxidant gene expression, similar findings were observed in common carp, where the antioxidant gene expressions were higher in the liver compared to the intestine. This may be attributable to the tissue-specific expression of antioxidant genes under oxidative stress. In carp, oxidative stress enhanced antioxidant gene transcription values in the liver, but reduced them in other tissues [113].

Aquaculture’s predominant purpose is to improve the maximum growth rate while maintaining the lowest feed conversion ratio [114]. A wide range of research has been undertaken to fulfil this purpose, and feed additives are one of the most promising ones [115,116]. Enhanced growth output and feed utilization in Nile tilapia fed SB were noticed in our study. The findings complied with earlier work in peninsula carp (*Labeo fimbriatus*) [117]; dairy cows [118]; and broilers [119]. SB has been shown to proliferate *Bacillus* spp. in the chicken’s intestinal tract, which enhances gut health and chicken performance [119]. Furthermore, SB has been considered to be a prebiotic source [28,29,120], known to boost fish growth and feed utilization [107,121].

Biofloc technology plays an essential part in decreasing feed utilization and stimulating the health and wellbeing of aquacultural species [38,39,40,122]. Previous studies have demonstrated that biofloc technology combined with functional feed additives significantly enhanced growth performance, immunity, and disease resistance [123,124,125]. Similar results were remarked in fish fed SB in our work. SB has been demonstrated to be a good source of fiber and a potential prebiotic [18,101,102,103]. Kishawy, Sewid, Nada, Kamel, El-Mandrawy, Abdelhakim, El-Murr, Nahhas, Hozzein, and Ibrahim [125] reported that mannan oligosaccharide (MOS–a prebiotic) administration to the biofloc system led to an increase in LAB population in the water and the intestine, modulated immune response and tolerance against *Aeromonas hydrophila*, and caused a rise in the survivability and performance of Nile tilapia. Sugarcane bagasse is a potential organic carbon source [126,127,128,129]. It is known that incorporation of MOS carbon sources into biofloc systems trigger heterotrophic microorganisms to take up the inorganic nitrogen, thereby modifying the water C:N ratio, resulting in greater microbic protein sources for host, as well as enhanced water quality [42,130]. Furthermore, the integration of MOS as a carbon source results in the development of biofloc, an additional protein source for fish [131]. Additionally, MOS serves as a means of carbon and is recognized as a prebiotic carbohydrate, which has been documented to boost growth efficiency by enhancing the augmentation of LAB in the fish intestine [132]. These favorable microorganisms are capable of releasing mannanase enzymes that metabolize MOS and generate fermented acids, like lactic and citric acids [133]. Hence, the dietary inclusion of SB may generate the same effects as MOS within the biofloc system, which boosts growth, immunity, and disease protection of the host.

## 5. Conclusions

The addition of sugarcane bagasse (SB) to tilapia diets raised in biofloc water boosted growth performance and skin mucosal and serum immunities, as well as enhancing immune-related and antioxidant gene expressions. SB seems to be an acceptable, ecologically responsible substance for improving Nile tilapia growth and health status.

## Figures and Tables

**Table 1:** The formulation and proximate composition of the experiment (g kg^−1^).

Ingredients	SB0	SB10	SB20	SB40	SB80
Fish meal	150	150	150	150	150
Corn meal	200	200	200	200	200
Soybean meal	390	390	390	394	400
Wheat flour	70	70	70	70	70
Rice bran	150	150	145	126	80
SB ^1^	0	10	20	40	80
Cellulose	20	10	5	0	0
Soybean oil	5	5	5	5	5
Premix ^2^	10	10	10	10	10
Vitamin C ^3^	5	5	5	5	5
Proximate composition of the experimental diets (%)					
Crude protein	32.6	32.4	32	31.4	31.8
Crude lipid	2.34	2.57	2.69	3.58	2.68
Fiber	3.75	3.83	4.35	4.76	5.06
Ash	7.75	7.72	7.67	7.50	7.34
Dry matter	96.63	96.80	94.02	93.97	96.8
GE (cal/g) ^4^	4239	4255	4200	4214	4219

^1^ SB = sugarcane bagasse; ^2^ vitamin and trace mineral mix supplemented as follows (IU kg^–1^ or g kg^–1^ diet): retinyl acetate 1,085,000 IU; cholecalciferol 217,000 IU; D, L-a-tocopherol acetate 0.5 g; thiamin nitrate 0.5 g; pyridoxine hydrochloride 0.5 g; niacin 3 g; folic 0.05 g; cyanocobalamin 10 g; Ca pantothenate 1 g kg^−1^; inositol 0.5 g; zinc 1 g; copper 0.25 g; manganese 1.32 g; iodine 0.05 g; sodium 7.85 g; ^3^ Vitamin C 98% 8 g; ^4^ GE = gross energy.

**Table 2:** Primer sequences, amplicons, and the related information for quantitative PCR.

Primer Name	Primer Sequence (5′-3′)	Target Gene	Tm (°C)	Product Size (bp)	Accession No.
18S rRNA -F	GTGCATGGCCGTTCTTAGTT	18S rRNA	60	150	XR_003216134
18S rRNA -R	CTCAATCTCGTGTGGCTGAA		60		
IL1-F	GTCTGTCAAGGATAAGCGCTG	IL-1	59	200	XM_019365844
IL1-R	ACTCTGGAGCTGGATGTTGA		58		
IL8-F	CTGTGAAGGCATGGGTGTG	IL-8	59	196	NM_001279704
IL8-R	ATCACTTTCTTCACCCAGGG		58		
LBP-F	ACCAGAAACTGCGAGAAGGA	LBP	59	200	XM_013271147
LBP-R	GATTGGTGGTCGGAGGTTTG		59		
GSTa-F	ACTGCACACTCATGGGAACA	GSTa	60	190	NM_001279635
GSTa-R	TTAAAAGCCAGCGGATTGAC		60		
GPX-F	GGTGGATGTGAATGGAAAGG	GPX	60	190	NM_001279711
GPX-R	CTTGTAAGGTTCCCCGTCAG		59		
GSR-F	CTGCACCAAAGAACTGCAAAC	GSR	60	172	XM_005467348
GSR-R	CAGAGAAGGCAGTCCACTC		60		

IL1: interleukin 1, IL8: interleukin 8, LBP: lipopolysaccharide binding protein, GSTa: glutathione S-transferase, GPX: glutathione peroxidase, GSR: glutathione-disulfide reductase.

**Table 3:** Growth performances and feed utilization (mean ± SE) of the Nile tilapia fed different diets: SB0 (0 -control), SB10 (10 g kg^−1^), SB20 (20 g kg^−1^), SB40 (40 g kg^−1^), and SB80 (80 g kg^−1^).

Ingredients	SB0	SB10	SB20	SB40	SB80
IW (g)	15.12 ± 0.007	15.12 ± 0.01	15.17 ± 0.01	15.10 ± 0.01	15.07 ± 0.004
FW (g)					
4 weeks	36.65 ± 0.21	36.23 ± 0.13	37.25 ± 0.11	37.43 ± 0.20	39.00 ± 0.25
8 weeks	71.48 ± 0.20 ^b^	71.35 ± 0.13 ^b^	74.78 ± 0.04 ^a^	75.60 ± 0.10 ^a^	71.90 ± 0.01 ^b^
WG (g)					
4 weeks	21.53 ± 0.20	21.12 ± 0.43	22.08 ± 0.12	22.33 ± 0.20	23.93 ± 0.25
8 weeks	56.83 ± 0.20 ^b^	56.23 ± 0.13 ^b^	59.62 ± 0.05 ^a^	60.50 ± 0.10 ^a^	56.83 ± 0.02 ^b^
FCR					
4 weeks	1.05 ± 0.006	1.08 ± 0.02	1.08 ± 0.002	1.04 ± 0.009	0.97 ± 0.009
8 weeks	1.23 ± 0.003 ^b^	1.23 ± 0.006 ^b^	1.22 ± 0.003 ^b^	1.18 ± 0.001 ^b^	1.29 ± 0.003 ^a^
SGR					
4 weeks	3.16 ± 0.02	3.11 ± 0.04	3.21 ± 0.01	3.24 ± 0.02	3.39 ± 0.02
8 weeks	2.77 ± 0.004 ^b^	2.77 ± 0.003 ^b^	2.85 ± 0.002 ^a^	2.88 ± 0.003 ^a^	2.79 ± 0.00 ^b^
SR (%)					
4 weeks	100	100	100	100	100
8 weeks	100	100	100	100	100

IW: initial fish weight, FW: final fish weight, WG: weight gain, SGR: specific fish growth rate^−1^, FCR: feed conversion ratio, SR: survival rate, SB: sugarcane bagasse. Different letters in a row denote significant difference (*p* < 0.05).

**Table 4:** Skin mucus lysozyme and peroxidase activities of *O. niloticus* after 4 and 8 weeks feeding with experimental diets: SB0 (0 -control), SB10 (10 g kg^−1^), SB20 (20 g kg^−1^), SB40 (40 g kg^−1^), and SB80 (80 g kg^−1^).

Ingredients	SB0	SB10	SB20	SB40	SB80
4 weeks					
SMLA	0.73 ± 0.08 ^b^	1.22 ± 0.06 ^ab^	1.64 ± 0.41 ^ab^	1.74 ± 0.43 ^a^	1.73a ± 0.17 ^a^
SMPA	0.08 ± 0.006 ^b^	0.09 ± 0.005 ^ab^	0.10 ± 0.003 ^a^	0.09 ± 0.005 ^ab^	0.10 ± 0.007 ^a^
8 weeks					
SMLA	1.50 ± 0.31 ^c^	2.89 ± 066 ^ab^	3.73 ± 0.95 ^a^	2.61 ± 0.90 ^b^	3.49 ± 1.19 ^ab^
SMPA	0.09 ± 0.006 ^b^	0.14 ± 0.006 ^a^	0.12 ± 0.001 ^a^	0.14 ± 0.003 ^a^	0.15 ± 0.02 ^a^

SMLA (μg mL^−1^) = skin mucus lysozyme activity; SMPA (μg mL^−1^) = skin mucus peroxidase activity. Different letters in a row denote significant difference (*p* < 0.05).

**Table 5:** Serum immunity of *O. niloticus* after four and eight weeks’ feeding with experimental diets: SB0 (0-Control), SB10 (10 g kg^−1^), SB20 (20 g kg^−1^), SB40 (40 g kg^−1^), and SB80 (80 g kg^−1^).

Ingredients	SB0	SB10	SB20	SB40	SB80
4 weeks					
SL	1.90 ± 0.29 ^c^	2.36 ± 0.02 ^bc^	3.20 ± 0.25 ^a^	2.87 ± 0.09 ^ab^	3.18 ± 0.35 ^a^
SP	0.22 ± 0.02	0.22 ± 0.07	0.28 ± 0.04	0.26 ± 0.04	0.21 ± 0.02
RB	0.12 ± 0.008 ^b^	0.21 ± 0.02 ^a^	0.10 ± 0.004 ^b^	0.14 ± 0.02 ^b^	0.12 ± 0.10 ^b^
8 weeks					
SL	5.69 ± 0.41 ^b^	8.68 ± 1.09 ^a^	8.42 ± 1.04 ^a^	8.72 ± 0.32 ^a^	7.88 ± 0.38 ^ab^
SP	0.19 ± 0.01	0.16 ± 0.01	0.17 ± 0.02	0.20 ± 0.02	0.17 ± 0.02
RB	0.25 ± 0.02	0.27 ± 0.006	0.18 ± 0.02	0.20 ± 0.005	0.25 ± 0.07

SL = serum lysozyme activity (μg mL^−1^); SP = serum peroxidase activity (μg mL^−1^); RB = respiratory burst activity (OD655). Different letters in a row denote significant difference (*p* < 0.05).

## Data Availability

The data that support the findings of this study are available from the corresponding author, [Van Doan, H.], upon reasonable request.
